# Protection of layers and breeders against homologous or heterologous HPAIv by vaccines from Korean national antigen bank

**DOI:** 10.1038/s41598-020-66343-9

**Published:** 2020-06-10

**Authors:** Yong-Myung Kang, Hyun-Kyu Cho, Hyun-Mi Kim, Chi-Ho Lee, Do-Young Kim, Sang-Hyun Choi, Myoung-Heon Lee, Hyun-Mi Kang

**Affiliations:** Animal and Plant Quarantine Agency, 177 Hyeoksin 8-ro, Gimcheon-si, Gyeongsangbuk-do, 39660 Republic of Korea

**Keywords:** Immunology, Microbiology

## Abstract

Korean government has selected and stocked five type antigens of two clades as Korean national antigen bank having high possibility of introduction to Korea. We aimed to evaluate the efficacy of the clade 2.3.2.1c and 2.3.4.4c H5Nx vaccines from the Korean avian influenza (AI) national antigen bank for emergency preparedness for their potency and protective efficacy against lethal homologous and heterologous viruses in layer and breeder chickens practically. The PD_50_ (dose of vaccine that protects 50% of chickens from viral challenge) of all vaccinated groups was >50, which was satisfied with minimum antigen requirement of OIE, and the PD_50_ levels of the two vaccines differed depending on strain and chicken breed. In homologous challenge, all vaccinated groups exhibited 100% survival with no clinical symptoms and high levels of pre-challenge protective immunity (7.2–8.5 log_2_), although they did not completely prevent virus shedding. On the other hand, against heterologous virus challenge, vaccinated animals exhibited 62.5–80% survival with lower antibody titers (2.3–3.4 log_2_) and a longer period of virus shedding (14 days post infection [dpi]). Our results suggest that the clade 2.3.2.1c and 2.3.4.4c H5Nx vaccines are good candidates for emergency vaccination of commercial chickens and support the idea that close genetic matching between vaccine and challenge virus provides the best protection.

## Introduction

An H5N1 highly pathogenic avian influenza (HPAI) A virus (A/Goose/Guangdong/1/96; Gs/GD/96) was first detected in China in 1996 and subsequently spread into Hong Kong in 1997, causing massive economic losses to the poultry industry^1,2^. Since 1997, multiple clades have evolved and spread across Asia, Africa, and Europe^3^. In Korea, H5Nx HPAI have been detected in both poultry farms and wild birds since 2003, including clades 2.5, 2.2, 2.3.2, 2.3.2.1, 2.3.4.4a, 2.3.4.4c, and 2.3.4.4b^4–8^.

In particular, HPAI outbreaks of two subtypes (H5N6 and H5N8) were reported in 343 and 76 poultry farms in 2016 and 2017, respectively. This period was associated with an unprecedented level of damage to the poultry industry in Korea: 38 million animals were culled, resulting in huge financial losses (approximately $312 million). AI vaccination in conjunction with surveillance and depopulation was required by some poultry producers and animal-welfare organizations. Accordingly, the Korean government has selected and stocked five types of antigens corresponding to two clades with a high risk of introduction into Korea, 2.3.2.1c and 2.3.4.4a, b, c and d (H5Nx), as a national AI antigen bank^9^.

Laboratory experiments related to inactivated vaccine development, using oil adjuvant in SPF (specific pathogen–free) chickens, have been conducted to assess correlates of vaccine efficacy such as prevention of mortality, reduction of infection rate, and reduction of viral shedding^10–12^. However, some studies reported that commercial poultry in the field do not achieve the same levels of vaccine efficacy as SPF chickens in the laboratory, due to multiple factors including age, housing environment, species, and immunization level^13–15^.

According to livestock rearing statistics from the Korean Statistical information Service (KOSIS), in 2019 a total of 175 million commercial chickens were raised in Korea on about 2,900 farms^16^. HPAI outbreaks have resulted in enormous economic damage to chicken farmers in this country^17^. Consequently, the main poultry targeted for emergency vaccination with vaccines in the national AI antigen bank are commercial chickens, including layers and breeders. In a previous study, we showed that vaccines from the national AI antigen bank were effective in SPF chickens^9^, but the practical effects of vaccines against HPAI in commercial chickens remained uncharacterized.

Hence, we sought to evaluate the efficacy of the clade 2.3.2.1c and 2.3.4.4c vaccines from the Korean national AI antigen bank against homologous and heterologous HPAI viruses (HPAIV) in layer and breeder chickens.

## Results

### Study 1: Potency of vaccines against homologous viruses in commercial chickens

#### Clinical protection

**Figure 1:**
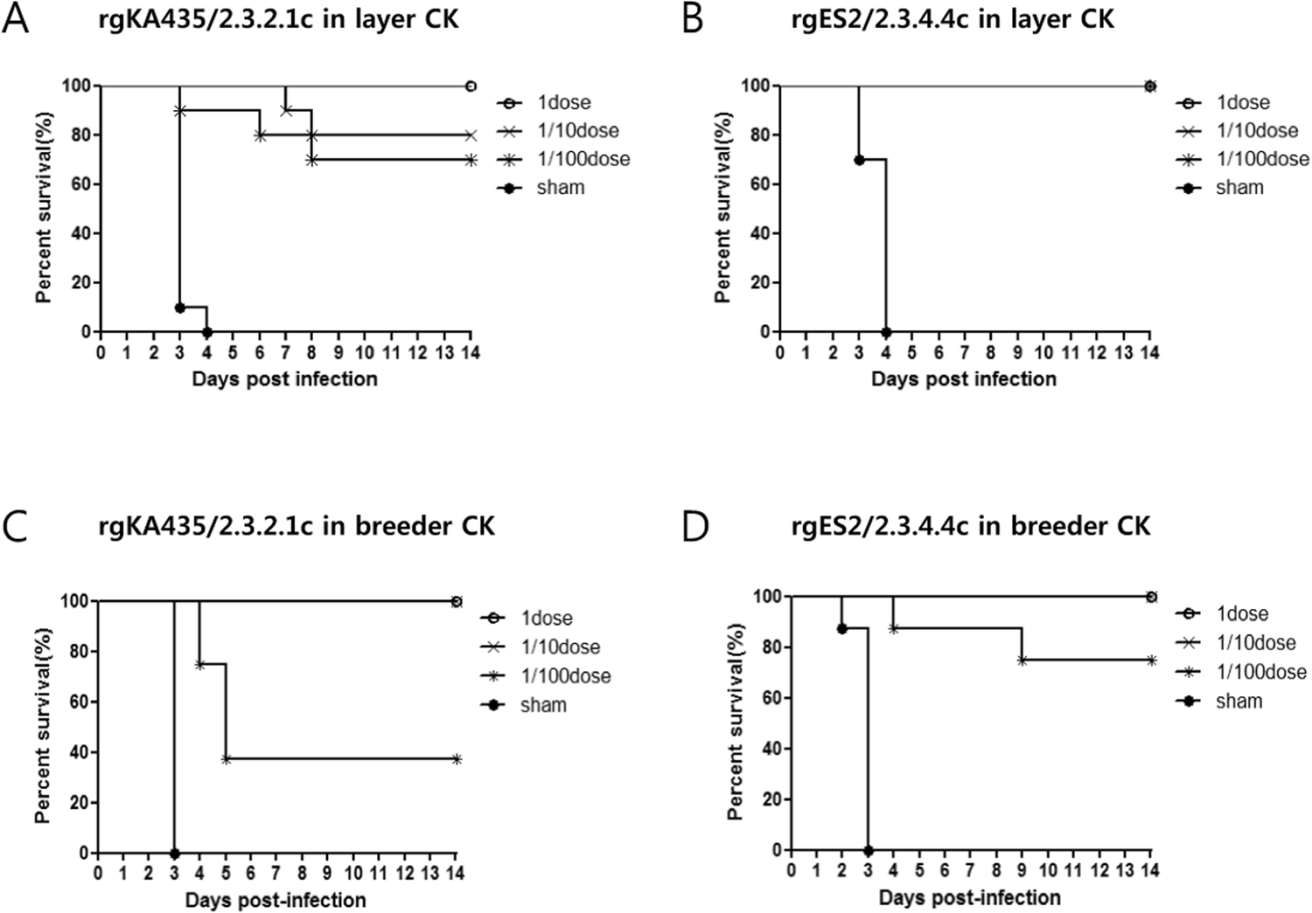
Survival of vaccinated chickens after challenge with homologous HPAIv. Survival of chickens inoculated with 1, 0.1, or 0.01 dose of one of two representative inactivated vaccines, or sham-vaccinated, followed by challenge with homologous HP H5 viruses. Vaccines were as follows: (**A**) rgKA435/2.3.2.1c in layer chickens, (**B**) rgES2/2.3.4.4c in layer chickens, (**C**) rgKA435/2.3.2.1c in breeder chickens, and (**D**) rgES2/2.3.4.4c in breeder chickens.

In layer and breeder chickens, vaccination with a 1 dose of rgKA435/2.3.2.1c conferred 100% clinical protection from challenge with homologous virus, with no clinical symptoms, whereas vaccination with 0.1 dose resulted in 20% mortality by 8 dpi only in layers (Fig. 1). Vaccination with 0.01 dose resulted in higher mortality and clinical signs of infection than the 1 dose and 0.1 dose groups. Vaccination of layer chickens with 0.01 doses led to 30% mortality by 8 dpi, with two chickens dying between 7 and 8 dpi with neurological signs and diarrhea (Fig. 1A). Vaccination of breeder chickens with 0.01 dose led to 60% mortality by 5 dpi (Fig. 1C), with four chickens dying between days 4 and 5 with neurological signs. For rgES2/2.3.4.4 C, vaccination with 0.01 dose resulted in no mortality in layer chickens (Fig. 1B), but 25% mortality in breeder chickens (Fig. 1D). The mean time to death (MDT) in both 0.01 dose vaccination groups was 4.6–6.0 days [Table 1]. For sham-treated chickens, mean time to death was 2.0–3.7 days.

**Table 1:** Results from vaccinations of two varieties of commercial chickens with varying doses of inactivated vaccines against HPAI.

Vaccines	Chicken species (age)	Antigen Dose^a^	Survival(%) (MDT)^b^	Morbidity	Peak shedding (3 dpi)^c^		HI titer(log_2_)^d^		PD_50_
					OP	CL	Pre-challenge (Homo)	Post-challenge (Homo)	
rgKA435/2.3.2.1c	Layer chickens (35w)	1	100		1/10 (0.1)	0/10 (−)	10/10 (7.5)	10/10 (8.7)	100
		1/10	80 (7.5)		6/10 (1.2)	2/10 (0.3)	10/10 (6.0)	10/10 (7.6)	
		1/100	70 (5.7)	Mild depression, green diarrhea	6/9 (1.3)	2/9 (0.3)	2/10 (3.3)	5/10 (7.1)	
		Sham	0 (3.1)	Lethargy, mortality	1/1 (3)	1/1 (3.3)	0/10 (−)	NT	
	Breeder chickens (35w)	1	100		0/8 (−)	0/8 (−)	8/8 (7.2)	8/8 (8.5)	63
		1/10	100		2/8 (0.4)	0/8 (−)	8/8 (6.5)	8/8 (7.6)	
		1/100	37.5 (4.6)	Mild depression, green diarrhea, mortality	8/8 (2.1)	7/8 (2.2)	5/8 (2.4)	3/3 (7.3)	
		Sham	0 (3)	Lethargy, mortality	NT	NT	0/8 (−)	NT	
rgES2/ 2.3.4.4c	Layer chickens (35w)	1	100		0/10 (−)	0/10 (−)	10/10 (8.5)	10/10 (9.6)	350
		1/10	100		1/10 (0.1)	0/10 (−)	10/10 (6.9)	10/10 (8.0)	
		1/100	100	Mild depression	0/10 (−)	0/10 (−)	7/10 (5.0)	10/10 (7.4)	
		Sham	0 (3.8)	Lethargy, mortality	7/7 (4.3)	6/7 (2.2)	0/10 (−)	NT	
	Breeder chickens (35w)	1	100		0/8 (−)	0/8 (−)	8/8 (7.5)	8/8 (8.3)	215
		1/10	100		2/8 (0.7)	0/8 (−)	8/8 (7.1)	8/8 (8.0)	
		1/100	75 (6.5)	Depression, green diarrhea, mortality	2/8 (0.7)	1/8 (0.3)	8/8 (4.8)	5/5 (6.6)	
		Sham	0 (2.9)	Lethargy, mortality	NT	NT	0/8 (−)	NT	

^a^One dose (1) contained 512 HAU (hemagglutination units).

^b^MDT = mean death time (days).

^c^No. virus positive/total in group (mean shed-virus titer).

^d^No. serology positive/total surviving in group (mean HI titer).

No. of commercial chicken: 10 layer chickens per group; 8 breeder chickens per group

Abbreviations: EID_50_, 50% egg infectious dose; HA, hemagglutination activity; NT, not tested; dpi, days post-infection; OP, oropharyngeal; CL, cloacal; HI, hemagglutination inhibition; PD_50_, 50% protective dose.

Clinical protection was also indicated by the vaccine potency results^18^. Potency values for layer and breeder chickens were higher in the rgES2/2.3.4.4c group (PD_50_ of 350 and 215, respectively) than in the rgKA435/2.3.2.1c group (PD_50_ of 100 and 63, respectively) [Table 1].

#### Serology

**Figure 2:**
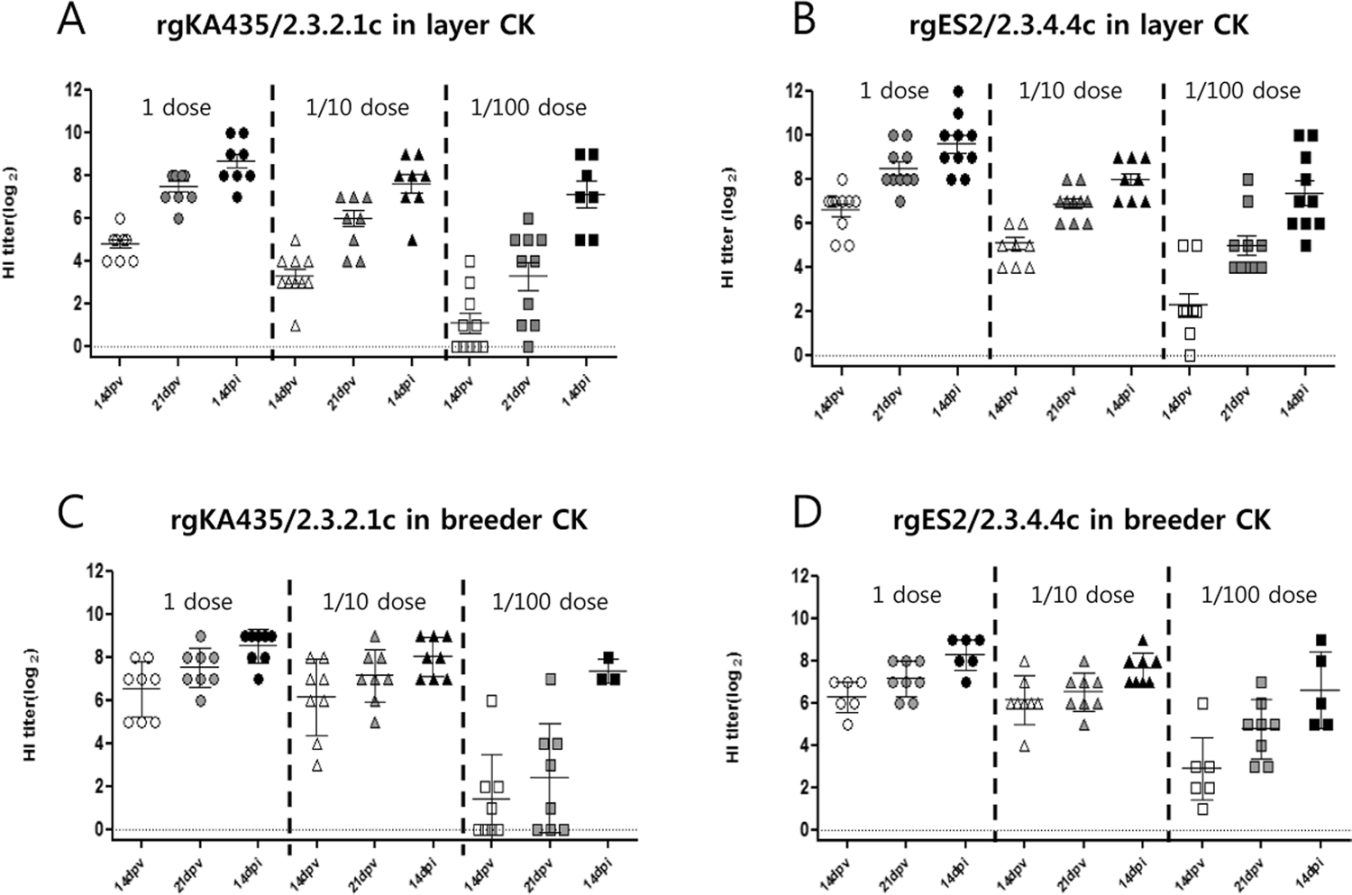
Serological response of chickens vaccinated and challenged. Hemagglutination inhibition (HI) assay titers in vaccinated chickens at the indicated times following vaccination and challenge with homologous virus. HI titers were assessed at 14 days post-vaccination (dpv), at 21 dpv, and at 14 days post-infection (dpi). Vaccines were administered at 1, 0.1, or 0.01 dose. Vaccines were (**A**) rgKA435/2.3.2.1c in layer chickens, (**B**) rgES2/2.3.4.4c in layer chickens, (**C**) rgKA435/2.3.2.1c in breeder chickens, and (**D**) rgES2/2.3.4.4c in breeder chickens. Individual data points are shown along with means and standard errors.

In all vaccinated groups, detectable antibody titers against homologous antigens were elevated both pre- and post-challenge (Fig. 2). Among the vaccinated (1 and 0.1 dose) chickens, those in the two representative vaccinated groups seroconverted before the challenge, with a mean titer of 7.2–8.5 and 6.0–7.1 log_2_ in the 1 dose and 0.1 dose groups, respectively. After challenge, antibody titer against homologous antigen increased to 8.3–9.6 and 7.6–8.0 log_2_ in the 1 dose and 0.1 dose groups, respectively [Table 1]. By contrast, in the 0.01 dose vaccinated groups, there were various antibody reaction showing HI positive with 2.4–5.0 log_2_ prior to challenge. Following challenge, HI titer of surviving chickens in all groups had more various antibody reactions showing HI positive with 6.6–7.4 log_2_. None of the sham-vaccinated chickens had detectable HI antibody titers before challenge (data not shown). Antibody titers against H9 antigen in both kinds of commercial chickens were 4.5–5.5 log_2_ pre-challenge. After challenge, antibody titers against H9 antigen did not differ between the two kinds of commercial chickens (data not shown).

#### Virus shedding

**Figure 3:**
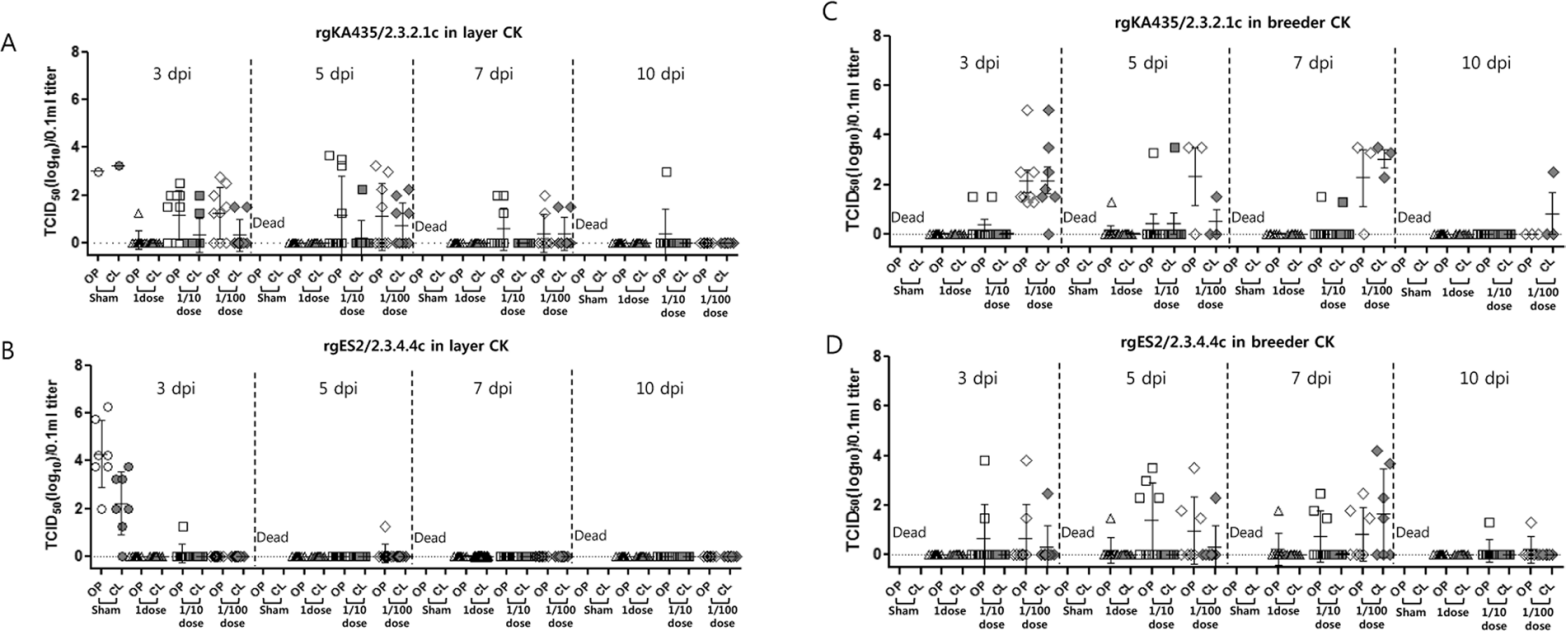
Virus shedding in oropharyngeal (OP) and cloacal (CL) swab samples after inoculation with homologous HPAIv. Titers of virus shed in OP and CL samples from chickens inoculated with inactivated vaccines (1, 0.1, or 0.01 dose, or sham-vaccinated), assessed at 3, 5, 7 and 10 days post-infection (dpi) with homologous HP H5 viruses. Viral titers are expressed as log_10_TCID_50_ (50% tissue culture infectious dose) in 0.1 ml, with error bars included. Vaccines were (**A**) rgKA435/2.3.2.1c in layer chickens, (**B**) rgES2/2.3.4.4c in layer chickens, (**C**) rgKA435/2.3.2.1c in breeder chickens, and (**D**) rgES2/2.3.4.4c in breeder chickens. The lower limit of detection was 1 log_10_TCID_50_ in 0.1 ml.

As shown in Fig. 3, little virus shedding was observed from 1–14 dpi in commercial chickens vaccinated with 1 dose. However, virus shedding was detected from 3–10 dpi in commercial chickens vaccinated with rgKA435/2.3.2.1c (0.1 dose) and rgES2/2.3.4.4c (0.1 dose), except in layer chickens vaccinated with rgES2/2.3.4.4c (0.1 dose) and breeder chickens vaccinated with rgKA435/2.3.2.1c (0.1 dose) (Fig. 3). Virus shedding was detected in surviving commercial chickens vaccinated with 0.01 dose, with a viral titer of 10^1.3^− 10^3.5^ TCID_50_/0.1 ml from 3–10 dpi in OP swab samples and 10^1.5^–10^3.0^ TCID_50_/0.1 ml from 3–10 dpi in CL swab samples (TCID_50,_ 50% Tissue Culture Infectious Dose). Most sham-vaccinated groups were not tested because they died before a swab was taken; however, in layer chickens of each sham-vaccinated group, virus shedding peaked within 3 dpi in surviving chickens: OP swab samples, 10^3.0^–10^4.3^ TCID_50_/0.1 ml; CL swab samples, 10^2.6^−10^3.3^ TCID_50_/0.1 ml.

### Study 2: Efficacy of vaccines against challenge with heterologous viruses

**Figure 4:**
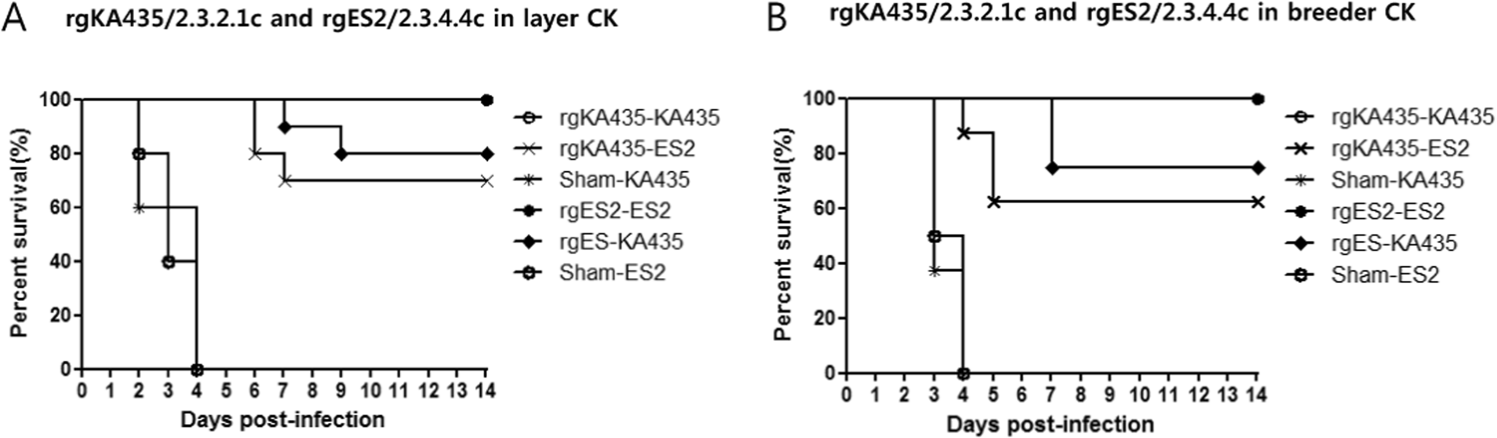
Survival of vaccinated chickens after challenge with homologous and heterologous HPAIv. Survival of chickens inoculated with one of two representative inactivated vaccines, or sham-vaccinated, followed by challenge with homologous or heterologous HP H5 viruses. Vaccines were as follows: (**A**) rgKA435/2.3.2.1c and rgES2/2.3.4.4c in layer chickens, followed by challenge with homologous or heterologous virus, and (**B**) rgKA435/2.3.2.1c and rgES2/2.3.4.4c in breeder chickens, followed by challenge with homologous or heterologous virus.

**Figure 5:**
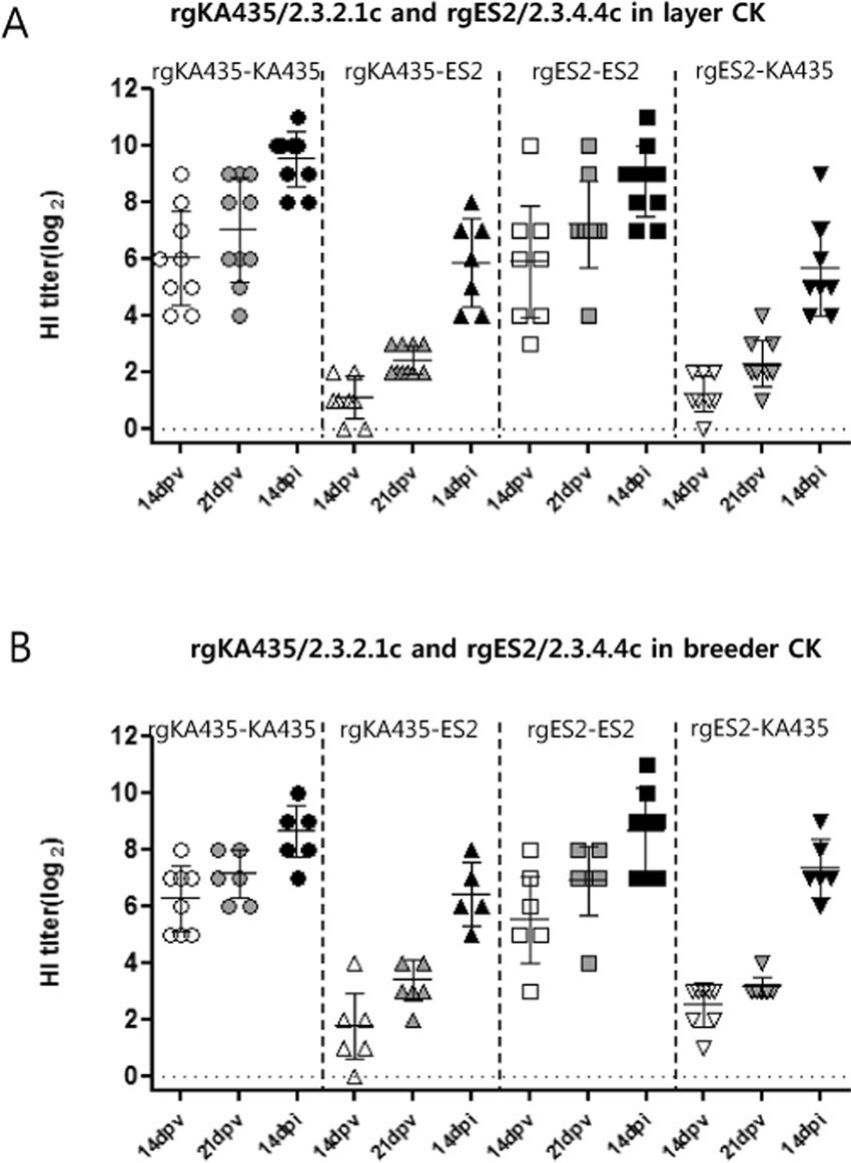
Serological response of chickens after vaccination and challenge. Hemagglutination inhibition (HI) assay titers in vaccinated chickens at the indicated times following vaccination and challenge with homologous and heterologous viruses. HI titers were assessed at 14 days post-vaccination (dpv), at 21 dpv, and at 14 days post-infection (dpi). Single (full) vaccine doses were administered. Vaccines were (**A**) rgKA435/2.3.2.1c and rgES2/2.3.4.4c in layer chickens, followed by challenge with homologous or heterologous virus, and (**B**) rgKA435/2.3.2.1c and rgES2/2.3.4.4c in breeder chickens, followed by challenge with homologous or heterologous virus. Individual data points are shown, along with means and standard errors.

**Figure 6:**
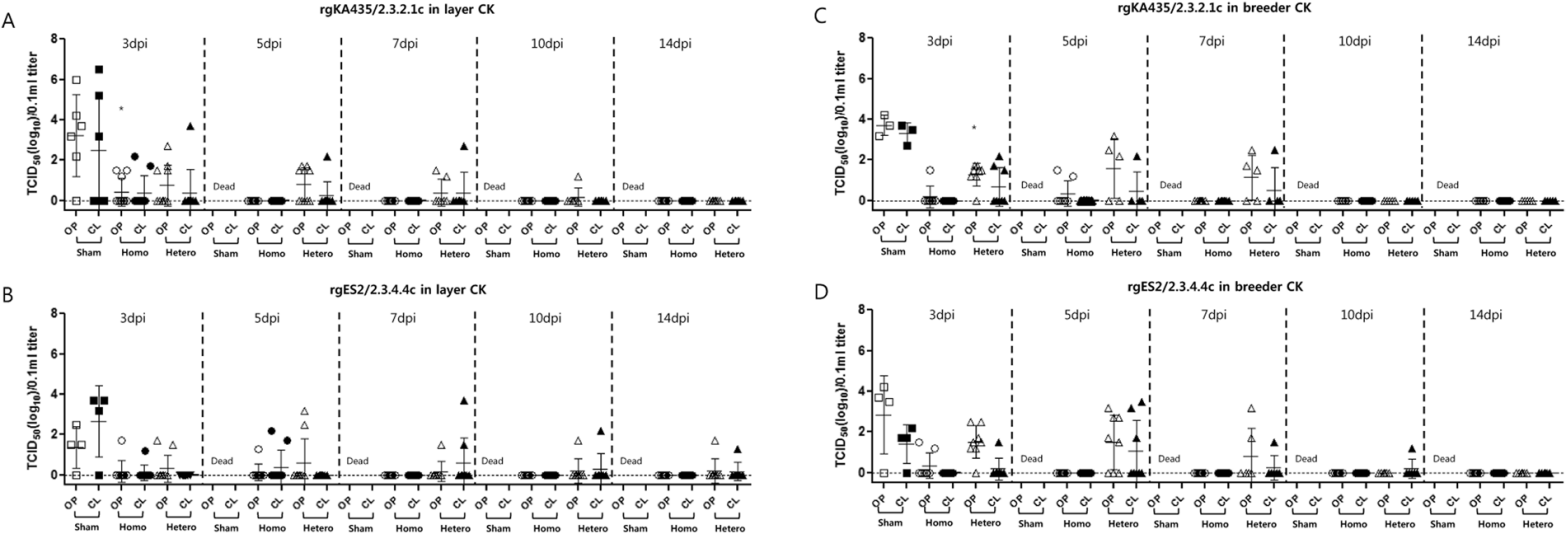
Virus shedding in oropharyngeal (OP) and cloacal (CL) swab samples after inoculation with homologous and heterologous HPAIv. Titers of virus shed in OP and CL samples from chickens inoculated with inactivated vaccines, assessed at 3, 5, 7, 10 and 14 days post-infection (dpi) with homologous and heterologous HP H5 viruses. Viral titers are expressed as log_10_TCID_50_ (50% tissue culture infectious dose) in 0.1 ml, with error bars included. Vaccines were (**A**) rgKA435/2.3.2.1c in layer chickens, followed by challenge with homologous and heterologous virus, (**B**) rgES2/2.3.4.4c in layer chickens, followed by challenge with homologous and heterologous virus, (**C**) rgKA435/2.3.2.1c in breeder chickens, followed by challenge with homologous and heterologous virus, and (**D**) rgES2/2.3.4.4c in breeder chickens, followed by challenge with homologous and heterologous virus. The lower limit of detection was 1 log_10_TCID_50_ in 0.1 ml.

We next examined the protective efficacy of vaccines in commercial chickens against the heterologous viruses KA435 and ES2. In commercial chickens vaccinated with rgKA435/2.3.2.1c and rgES2/2.3.4.4c, survival rate was 100% following challenge with homologous virus, but 62.5–80% following challenge with heterologous viruses (Fig. 4). All vaccinated groups had low detectable antibody titers against the corresponding heterologous antigen both pre- and post-challenge (Fig. 5). All chickens in the two representative vaccinated groups seroconverted before the challenge, with a mean titer of 2.3–3.4 log_2_ against heterologous antigen. After challenge, antibody titer against heterologous antigen increased to 5.6–7.3 log_2_ [Table 2]. After challenge with heterologous viruses, virus shedding was detected from 3–14 dpi in surviving chickens, with a viral titer of 10^1.3^− 10^2.9^ TCID_50_/0.1 ml in OP swab samples and 10^1.3^–10^3.7^ TCID_50_/0.1 ml from 3–14 dpi in CL swab samples (Fig. 6). Significant differences (*p* < *0.05*) between viral titers at 3 dpi in sham-vaccinated chickens were identified in both types of commercial chickens vaccinated with rgKA435/2.3.2.1c (Fig. 6A,C). In layer chickens vaccinated with rgKA435/2.3.2.1c and challenged with heterologous virus, virus shedding was detected by 14 dpi in one chicken, with viral titers of 10^1.7^ and 10^1.3^ TCID_50_/0.1 ml in OP and CL samples, respectively (Fig. 6B).

**Table 2:** Results from vaccinations of two varieties of commercial chickens with inactivated vaccines against homologous and heterologous HPAI.

Chicken species (age)	Vaccine strain (antigen dose^a^)	Challenge Strain^b^	Survival (%) (MDT)^c^	Peak shedding (3 dpi)^d^		HI titer (log_2_)^e^	
				OP	CL	Pre-challenge	Post-challenge
Layer chickens (35w)	rgKA435/2.3.2.1c (one dose)	KA435	100 (−)	3/10 (0.4)	2/10 (0.4)	10/10(7.0)	10/10(9.5)
		ES2	70 (6.3)	4/10 (0.7)	1/10 (0.4)	10/10(2.4)	7/7(5.9)
	Sham	KA435	0 (3.2)	5/6 (3.2)	3/6 (2.5)	0/10(−)	0/0(−)
	rgES2/2.3.4.4c (one dose)	ES2	100 (−)	1/10 (0.2)	1/10 (0.1)	10/10(7.2)	10/10(8.7)
		KA435	80 (8.5)	2/10 (0.3)	0/10 (−)	10/10(2.3)	8/8(5.6)
	Sham	ES2	0 (4.0)	3/4 (1.4)	3/4 (2.7)	0/10(−)	0/0(−)
Breeder chickens (35w)	rgKA435/2.3.2.1c (one dose)	KA435	100 (−)	1/8 (0.2)	0/8 (−)	8/8(7.1)	8/8(8.6)
		ES2	62.5 (4.7)	7/8 (1.3)	3/8 (0.7)	8/8(3.4)	5/5(6.4)
	Sham	KA435	0 (3.4)	3/3 (3.7)	3/3 (3.3)	0/8(−)	0/0(−)
	rgES2/2.3.4.4c (one dose)	ES2	100 (−)	2/8 (0.3)	0/8 (−)	8/8(7.3)	8/8(8.6)
		KA435	75 (7.0)	7/8 (1.5)	1/8 (0.2)	8/8(3.1)	6/6(7.3)
	Sham	ES2	100	3/4 (2.9)	3/4 (1.4)	0/8(−)	0/0(−)

^a^One dose (1) contained 512 HAU (hemagglutination units).

^b^Homologous: same as vaccine strain; heterologous: different from vaccine strain.

^c^MDT = mean death time (days).

^d^No. virus positive/total in group (mean shed-virus titer).

^e^No. serology positive/total survived in group (mean HI titer).

No. of commercial chickens: 10 layer chickens per group; 8 breeder chickens per group

Abbreviations: EID_50_, 50% egg infectious dose; HA, hemagglutination activity; NT, not tested; dpi, days post-infection; OP, oropharyngeal; CL, cloacal; HI, hemagglutination inhibition; PD_50_, 50% protective dose.

## Discussion

It is crucial that vaccine potency and efficacy are evaluated in commercial chickens prior to their emergency use in the field, because the efficacy of AI vaccines differs among poultry species (chicken, duck, and quail) and breeds (layer and breeder)^19^. Hence, we evaluated vaccine efficacy against homologous and heterologous HPAIV in commercial chickens by measuring antibody titers after vaccination and monitoring clinical signs and virus shedding post-infection.

The minimum antigen requirement for licensing vaccines is 50 PD_50_ per dose, which ensures that there is sufficient antigen mass or virus titer to be efficacious in the field^20,21^. The two representative vaccines that we obtained from the Korean national AI antigen bank satisfied this criterion. The PD_50_ values of rgES2/2.3.4.4c in layers and breeders were higher than those of rgKA435/2.3.2.1c. Moreover, rgES2/2.3.4.4c yielded a 100% survival rate, depending on dose (1 to 0.01), and no virus shedding was observed in OP and CL at 3 dpi except in one layer chicken that received 0.1 dose [Table 1]. The differences in PD_50_ among vaccine strains may be attributed to differences in the virulence of homologous challenge viruses, despite their similar immunogenicity. This result corresponds with a report that one virus within clade 2.3.2.1c^22^ had a higher Lethal Dose 50 (LD_50_) and shorter Mean Death Time (MDT) in chickens than ES2/2.3.4.4c^23^. Finally, differences in pathogenicity between viruses result in differences in mortality among vaccinated chickens, again depending on the dose, and were reflected in the PD_50_ value^18^.

Antibody titers in commercial chickens are lower than those in SPF chickens due to average 35 weeks age and puberty. However, in previous study, two representative vaccines from the national AI antigen bank had higher potency in both kinds of commercial chickens than in SPF chickens^9^. The layer chickens used in this study were 35-week-old and had brown feathers, which are recognized as exhibiting reduced virulence against HPAI^24^. Additionally, the pathogenesis of avian influenza might be showed in the difference of susceptibility depending on age in various reports^25–29^ although it was recently reported that age is not a determinant factor in susceptibility to H5N2 HPAIv^30^. Our results are not consistent with a previous study showing that commercial chickens experienced less immunization than laboratory chickens due to maternal antibodies, immunosuppressive viruses, and the use of a lower vaccine dose^31^.

For emergency vaccination, it was important to evaluate cross-protection against heterologous viruses in commercial chicken. Although they did not completely prevent virus shedding in commercial chickens, the two vaccine groups exhibited a 100% survival rate and higher antibody titer against homologous challenge. By contrast, heterologous vaccine groups yielded a 62.5–80% survival rate with lower HI titer. This result corresponds with a previous report that protection in low antibody titer might be satisfied minimum antibody titer [> 3 log_2_] for survival in case the vaccine and field viruses^32^. Meanwhile, some layer chickens under 3 log_2_ HI titer could survive challenge with heterologous virus, possibly due to their H9N2 antibody titer of 4.5–5.5 log_2_. Seo et. al^33^. reported that most young chickens infected with an H9N2 influenza virus survived lethal challenge with an H5N1 influenza virus, but infected birds shed H5N1 influenza virus in their feces due to adoptive transfer of T lymphocytes or CD8 + T cells. However, further study is needed on how H9N2 vaccination affects H5 HPAI vaccination to host.

Notably, virus shedding in OP and CL was observed until 14 dpi in one breeder chicken (Fig. 6B). This could be due to the distinguishable amino acidic differences (10.5%, data not shown) in whole hemagglutinin (HA) similarity between KA435/2.3.2.1c and ES2/2.3.4.4c. Antigenic matching of HA between vaccine and field viruses provides the best protection against mortality and virus shedding, assuming a comparable host immune response^34^. Therefore, emergency vaccination should only be considered if the vaccine is a 95% or better match to the strains circulating in Korea.

In conclusion, our study demonstrated the potency and efficacy of two representative vaccines, clade 2.3.2.1c and 2.3.4.4c H5Nx, and confirmed that they offered good protection against homologous challenge in commercial chickens. In addition, we found that vaccine potency may be influenced by the virulence of the challenge virus, as well as chicken breed. Cross-protection testing revealed that survival rate was lower, and virus shedding period was longer, when the vaccine and field strain were mismatched. Our findings suggest that these two representative vaccines effectively protect commercial chickens against homologous viruses, but are significantly less protective when the vaccine and field strain are mismatched.

## Materials and Methods

### Viruses and vaccine development

Two different H5 HPAIVs were used as inactivated-vaccine seed strains and challenge strains. These strains were selected from the Korean national AI antigen bank. Two of them, A/duck/Korea/ES2/2016 (H5N6 clade 2.3.4.4c)^7^ (hereafter ES2/2.3.4.4c) were isolated from a poultry farm in Korea, whereas A/chicken/Vietnam/NCVD-KA435/13 (H5N1 clade 2.3.2.1c)^9^ (hereafter KA435/2.3.2.1c) was kindly provided by the National Center for Veterinary Diagnostics in Vietnam. The viruses were propagated for 60 h in 10-day-old embryonated eggs of specific-pathogen–free (SPF) chickens. Two representative vaccine candidate strains (ES2/2.3.4.4c and KA435/2.3.2.1c) were obtained using a plasmid-based reverse genetics system based on v2pHW^35^, as prepared in previous study^9^.

### Animals (layer and breeder chickens)

Vaccine efficacy and potency experiments used layer(Hy-line brown) and breeder(Ross) chickens obtained from Korean commercial chicken farms. Specifically, the animals were 35-week-old layer and breeder chickens serologically positive for H9 due to H9N2 LPAI vaccination and negative for H5, as determined by the hemagglutination inhibition (HI) assay. All experiments with live H5 virus were performed in biosafety level 3 facilities, following guidelines approved by the Animal Ethics Committee of the Animal and Plant Quarantine Agency, Korea (Approval number: 2019–176).

### Study 1: Potency of vaccines in commercial chickens against homologous viruses

To evaluate the potency (in terms of PD_50_, the dose of vaccine that protects 50% of chickens from viral challenge) and efficacy of the inactivated vaccines, 40 35-week-old layer chickens and 32 35-week-old breeder chickens for each vaccine were divided into four groups (10 chickens per group in layers; 8 chickens per group in breeders): three immunization groups and one non-immunization (sham) group. Immunizations, which were intramuscular, delivered 1, 0.1, or 0.01 doses, obtained by serially diluting the vaccines in PBS and mixing the dilutions (30:70, w/w) with the adjuvant Montanide ISA VG70. The sham group was inoculated with PBS and the adjuvant Montanide ISA VG70. At 3 weeks post-vaccination (wpv), chickens were challenged intranasally with 0.1 ml of PBS containing 10^6^ EID_50_ (the amount of virus that will infect 50 percent of inoculated eggs) of virus homologous to the vaccine strain. Post-challenge, chickens were monitored daily for clinical signs and survival. PD_50_ was calculated using mortality as the endpoint, as described previously^36^.

#### Serology and antibody assays

Serum samples were collected from all chickens prior to vaccination and weekly for 3 weeks following vaccination. Blood samples were also obtained from all living chickens at 14 days post-challenge. Hemagglutination inhibition (HI) assays were performed using standard methods and homologous antigens (8 HA units, as determined using chicken RBCs)^37^.

#### Post-challenge virus shedding

Oropharyngeal and cloacal swabs were collected from animals in all groups at 3, 5, 7, 10, and 14 days post-challenge (dpc). Each oropharyngeal or cloacal sample was suspended in 1 ml of maintenance medium containing antibiotic–antimycotic mixture (Invitrogen, Carlsbad, CA, USA). Samples were used for inoculation of Dermal Fibroblast 1 (DF1) cells, and virus growth was determined based on cytopathic effects (CPE) and HA activity. Virus titers were calculated as described elsewhere^36^, and the limit of virus detection was <1. Statistical significance of differences between measurements was determined using Student’s *t*-test, with a *P*-value <0.05 indicating a significant difference.

### Study 2: Efficacy of vaccines in commercial chickens against challenge with heterologous vaccines

To evaluate the efficacy of two representative vaccines (rgKA435/2.3.2.1c and rgES2/2.3.4.4c) against challenge with heterologous viruses, 60 35-week-old layer chickens and 48 35-week-old breeder chickens for each vaccine were divided into six groups (10 chickens per group in layers and 8 chickens per group in breeders): four immunized and two non-immunized groups. The immunized groups were intramuscularly vaccinated with a single dose of rgKA435/2.3.2.1c or rgES2/2.3.4.4c vaccine with adjuvant Montanide ISA VG70. The two sham groups were inoculated with a mixture of PBS and adjuvant Montanide ISA VG70. To assess immunogenicity post-vaccination, all chickens in each group were bled weekly, and the HI assay was used to measure serum antibody levels in each group using homologous and heterologous antigens. Three weeks after vaccination, chickens were intranasally challenged with 10^6.0^ EID_50_/0.1 ml of homologous or heterologous strain (KA435/2.3.2.1c and ES2/2.3.4.4c). The protective efficacy of the vaccine was determined by evaluating clinical signs, mortality, and virus shedding after intranasal challenge with homologous and heterologous strains. Oropharyngeal and cloacal swabs were collected from animals in all groups at 3, 5, 7, 10, and 14 days post-challenge (dpc). Samples were used for inoculation of cultures of DF1 cells, and virus growth was determined based on cytopathic effects (CPE) and HA activity as described previously.

### Statistical analysis

Data were analyzed using the Prism version 5.0 software (GraphPad Software, La Jolla, CA, USA). Comparisons of serum titers between groups were made by one-way analysis of variance (ANOVA). Survival rates among groups were analyzed using the log–rank test. *P* < 0.05 was interpreted as statistically significant.

### Ethical approval

All experimental methods were conducted in accordance with relevant guidelines and regulations approved by OIE terrestrial Manual and, Animal and Plant Quarantine Agency, Korea (Approval number: 2019–176).
